# Hepatoprotective properties of *Penthorum chinense* Pursh against carbon tetrachloride-induced acute liver injury in mice

**DOI:** 10.1186/s13020-017-0153-x

**Published:** 2017-10-30

**Authors:** Meng Wang, Xiao-Jiao Zhang, Ruibing Feng, Yun Jiang, Da-Yong Zhang, Chengwei He, Peng Li, Jian-Bo Wan

**Affiliations:** 1 State Key Laboratory of Quality Research in Chinese Medicine, Institute of Chinese Medical Sciences, University of Macau, Room 6034, Building N22, Avenida da Universidade, Macao, People’s Republic of China; 2 Sichuan New Lotus Traditional Chinese Herb Limited Company, Chengdu, China

**Keywords:** Carbon tetrachloride, Nuclear factor E2-related factor 2, Hepatotoxicity, Oxidative stress, *Penthorum chinense* Pursh

## Abstract

**Background:**

*Penthorum chinense* Pursh (Penthoraceae, PCP), a well-known Miao ethnomedicine, has been traditionally used to treat several liver-related diseases, such as jaundice and viral hepatitis. The aims of the present study were to evaluate the probable properties of the aqueous extract of PCP on carbon tetrachloride (CCl_4_)—induced acute liver injury in mice.

**Methods:**

C57BL/6 mice were orally administered an aqueous extract of PCP (5.15 and 10.3 g/kg BW) or silymarin (100 mg/kg) once daily for 1 week prior to CCl_4_ exposure. Silymarin serves as a positive drug to validate the effectivenes of PCP.

**Results:**

A single dose of CCl_4_ exposure caused severe acute liver injury in mice, as evidenced by the elevated serum levels of alanine aminotransferase (ALT), aspartate aminotransferase (AST) and alanine phosphatase (ALP), and the increased TUNEL-positive cells in liver, which were remarkably ameliorated by the pretreatment of PCP. PCP was also found to decrease the levels of malondialdehyde (MDA), restore the glutathione (GSH) and enhance the activities of superoxide dismutase (SOD) and catalase (CAT) in the liver. In addition, the pretreatment of PCP inhibited the degradation of hepatic cytochrome P450 2E1 (CYP2E1), up-regulated the expression of nuclear factor erythroid 2-related factor 2 (Nrf2) and its target proteins in CCl_4_-treated mice.

**Conclusion:**

Results indicated that the pretreatment of PCP (10.3 g/kg BW) effectively protected against CCl_4_-induced acute liver injury, which was comparable to efficacy of silymarin (100 mg/kg). This hepatoprotective effects might be attributed to amelioration of CCl_4_-induced oxidative stress via activating Nrf2 signaling pathway.

**Electronic supplementary material:**

The online version of this article (10.1186/s13020-017-0153-x) contains supplementary material, which is available to authorized users.

## Background

Oxidative stress is well known to be involved in the pathogenesis of acute or chronic liver injury [1]. The over generation of reactive oxygen species (ROS) may be induced by various hepatotoxicants, including heavy metals, alcohol and carbon tetrachloride (CCl_4_) [2]. As a chemical inducer, CCl_4_ has been extensively used to assess the protection of natural products on liver injury in experimental cellular and animal models [3]. CCl_4_ is metabolized in the liver by cytochrome P450 2E1 (CYP2E1), and mainly yields the highly-reactive trichloromethyl radicals, which disturbs the redox homeostasis and causes oxidative stress. These free radicals may cause cellular DNA damage and the increased lipid peroxidation by reacting with cellular unsaturated lipids, leading to hepatocyte apoptosis and necrosis, which ultimately results in liver injury [4].

Antioxidant defense system, including non-enzymatic and enzymatic mechanisms, is principally responsible for protecting living organism from oxidative stress [5]. Among them, superoxide dismutase (SOD), catalases (CAT) and glutathione peroxidase (GSH-Px) serve as three main classes of antioxidant defense-related enzymes, which are modulated by nuclear factor-erythroid 2-related factor-2 (Nrf2) [6]. Normally, Nrf2 is restrained in cytosol through interacting with Kelch-like ECH-associated protein 1 (Keap1), a specific repressor [7]. Upon oxidative stress, Nrf2 translocates to the nucleus after the dissociation with Keap1, and regulates the expressions of antioxidant-related genes, including haem oxygenase 1 (HO-1) and glutamate cysteine ligase (GCL) [8]. Thus, chemicals or natural products that can activate Nrf2 signaling pathway might be used to prevent CCl_4_-induced liver injury.

*Penthorum chinense* Pursh (*Penthoraceae*, PCP), has been traditionally used as the Miao ethnomedicine and folk remedy in the treatment of liver-related diseases, including jaundice and viral hepatitis for a long time [9]. PCP is widely cultivated in Gulin county, Sichuan Province, China, around where there are a lot of liquor factories. The tea made from the aerial of PCP became more popular in local residents who often drink liquor, and the bartenders who work in liquor factories. In the recent years, a number of studies have demonstrated that PCP or its ingredients possess the diverse bioactivities, including antioxidant, anti-complement, anti-hyperglycemic, and anti-hepatocarcinoma [10]. Our previous studies have also indicated that the aqueous extract of PCP could protect against both acute [11] and chronic alcohol-induced liver injury [12]. However, the impacts of PCP against CCl_4_-induced liver injury has not been adequately addressed. Therefore, in this study, we aimed to evaluate the probable protective properties of PCP against acute liver injury induced by CCl_4_, and further elucidate its underlying mechanisms with respect to Nrf2-mediated antioxidant response.

## Methods

### Materials and sample preparation

Aerial part of PCP was provided by Sichuan New Lotus Traditional Chinese Herb Limited Company (Chengdu, China). Its botanical origin was identified by Dr. Chun-Feng Qiao from our university. The aqueous extract of PCP was prepared as previously described [11, 12]. The dried powder of PCP (150 g) was decocted three times with 1500 mL water for 2 h each. After combination and filtration, the decoction was lyophilized by a freeze dryers (VirTis BenchTop Pro, SP Scientific, Warminster, PA, USA). The freeze-dried extract was re-constructed by the distilled water for the present animal study. To assure the repeatability of the pharmacological study, pinocembrin-7-O-β-d-glucoside, the chemical marker, was measured as 3.49 mg/g in raw PCP by HPLC–UV. The voucher specimen of PCP sample (No. GHX201401) was stored at Institute of Chinese Medical Sciences, University of Macau, Macau.

### Animals and treatments

Mice (C57BL/6, 8–9 weeks old) were housed in institutional individually ventilated cage (IVC) system. All animals were assigned randomly to five groups (n = 10, half males and half females in each group), i.e. control group, CCl_4_ group, silymarin-treated group (100 mg/kg BW, as the positive control), two PCP-treated groups (5.15 and 10.3 g/kg BW). The dose of PCP (10.3 g/kg) was calculated from the usage of *Gan*-*Su*-*Ke*-*Li* (WS3-B-2526-97), a drug approved by China Food and Drug Administration (CFDA), which was made from the aqueous extract of PCP for the management of viral hepatitis. Mice were gavaged silymarin or PCP once daily for 1 week prior to CCl_4_ challenge in treatment group. 24 h after last dosing, animals were intraperitoneally injected with 10% CCl_4_ diluted in olive oil (*v/v*, 2 mL/kg) to induce acute liver injury [13], mice in the control group were treated with same volume of vehicle (*i.p.*). After fasting for 12 h, all mice were anesthetized, and serum samples and the entire liver tissues were immediately collected. Animal protocol was conducted according to the animal procedure approved by Animal Ethics Committee, Institute of Chinese Medical Sciences, University of Macau (ICMS-AEC-2015-05). The Minimum Standards of Reporting Checklist (Additional file 1) contains details of the experimental design, and statistics, and resources used in this study.

### Measurements of haematological parameters

Serum aspartate aminotransferase (AST), alanine aminotransferase (ALT) and alanine phosphatase (ALP) were examined through enzymatic colorimetric methods by their respective commercial assay kits (Nanjing Jiancheng Bioengineering Institute, Nanjing, China) according to the manufacturer’s instructions. For the measurements of AST and ALT, serum was mixed well with corresponding matrix solution, and then reacted with 2,4-dinitro-phenylhydrazine for 20 min. NaOH solution (4 mol/L) was added to terminate the reaction. Results were measured by SpectraMax® M5 Multi-Mode Microplate Reader (Waltham, MA, USA).

### Histopathological analysis

The right lobe of the liver tissue was fixed in 10% (*v/v*) phosphate-buffered formalin overnight, and embedded in paraffin. The cryostat sections were stained with hematoxylin and eosin according to a standard protocol [14]. The histopathological alterations of liver were observed by an Olympus CX-31 light microscopy with CCD camera (Olympus Crop, Tokyo, Japan).

### Parameter measurements for oxidative stress in the liver

Partial liver tissues were homogenized in 9 volumes of cold RIPA (Beyotime Institute of Biotechnology, Nanjing, China) on ice. The liver homogenates (10%) were centrifuged, and the final supernatants were subjected to measure the levels of malondiadehyde (MDA), reduced glutathione (GSH), oxidized glutathione (GSSG), the activities of superoxide dismutase (SOD) and catalase (CAT) by their respective assay kits (Jiancheng Bioengineering). Protein content in the homogenates was determined using a Pierce™ BCA Protein Assay Kit (Thermo Fisher Scientific Inc., Rockford, IL, USA). The results were normalized per gram total protein.

### TUNEL assay

The apoptotic cells in liver cryostat section were evaluated by a commercial ApopTag^®^ Plus In Situ Apoptosis Fluorescein Detection Kit (EMD Millipore Corporation, Billerica, MA, USA). Briefly, liver sections (4 μM) were fixed in 1% paraformaldehyde solution, then incubated in green fluorescein labelled dUTP solution at 37 °C for 1 h. After washing, the sections were counterstained with 4,6-diamidino-2-phenylindole (DAPI) (Thermo Fisher Scientific Inc., Rockford, IL, USA). The apoptotic cells were visualized on Zeiss Axio Imager A2 microscope (Carl Zeiss, Oberkochen, Germany).

### RT-PCR analysis

The transcriptional expressions of CYP2E1, Keap1, HO-1 and GCLC in liver were determined by qPCR as previously described [5, 11]. In brief, total RNA was extracted from liver by a TRIzol® reagent, and subjected to cDNA synthesis using TaqMan Reverse Transcription Reagents Kit (Life Technologies, Carlsbad, CA). The primers (Table 1) were synthesized by Invitrogen Life Technologies (Shanghai, China). qPCR was conducted on a Mx3005P qPCR system (Agilent Technologies) by SYBR^®^ Green PCR Master Mix (Life Technologies). The mRNA expression was normalized to β-actin.

**Table 1:** Primers used for the quantitative RT-PCR analysis

Gene	Forward primer	Reverse primer
CYP2E1	5′-CGTTGCCTTGCTTGTCTGGA-3′	5′-AAGAAAGGAATTGGGAAAGGTCC-3′
Keap1	5′-TGCCCCTGTGGTCAAAGTG-3′	5′-GGTTCGGTTACCGTCC TGC-3′
HO-1	5′-AAGCCGAGAATGCTGAGTTCA-3′	5′-GCCGTGTAATATGGTACAAGGA-3′
GCLC	5′-GGGGTGACGAGGTGGAGTA-3′	5′-GTTGGGGTTTGTCCTCTCCC-3′
β-actin	5′-GGCTGTATTCCCCTCCATCG-3′	5′-GTTGGGGTTTGTCCTCTCCC-3′

### Immunoblot analysis

The total protein was isolated from the left lobe of the liver tissues by the cold RIPA lysis buffer containing 1% phosphatase inhibitor cocktail (Beyotime Institute of Biotechnology). Approximately 60 μg of total proteins were loaded onto 10% SDS-PAGE, subsequently transblotted onto PVDF membranes (Bio-Rad Laboratories Inc., Hercules, CA, USA). After blocking with 5% nonfat dry milk in TBST (0.1% Tween-20 in Tris-buffered saline), the membranes were incubated with primary antibodies for 24 h at 4 °C, including CYP2E1 (1:1000, Cell Signaling Technology, Danvers, MA, USA), Keap-1 (1:1000, Santa Cruz Biotechnology, Santa Cruz, CA, USA), Nrf2 (1:200, Santa Cruz Biotechnology), HO-1 (1:250, Abcam, Cambridge, MA, USA), GCLC (1:1000, Abcam) and GAPDH (1:1000, Cell Signaling), then incubated with secondary antibodies conjugated to horseradish peroxidase (HRP) for 1 h. The proteins were visualized by Amersham ECL Select Western Blotting Detection Reagent (GE Healthcare BioSciences, Piscataway, NJ, USA).

### Statistical analysis

The value was expressed as mean ± SD. After verifying the distribution of the data by Kolmogorov–Smirnov test, the between-group comparison was conducted by ordinary one-way analysis of variance (ANOVA) using GraphPad 5.0 Software (San Diego, CA, USA).

## Results

### Effects of PCP on serum parameters

**Fig. 1:**
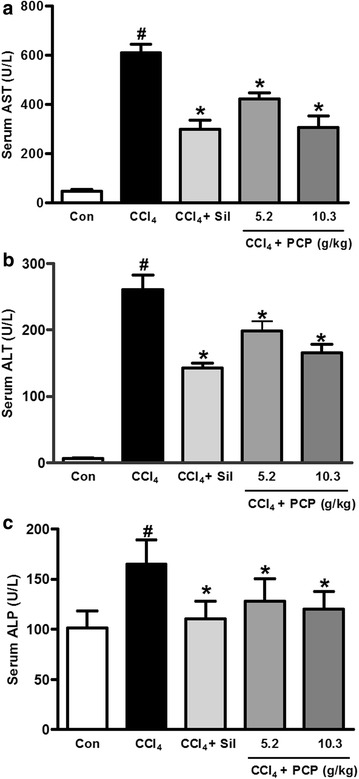
Effects of PCP on serum activities of **a** aspartate aminotransferase (AST), **b** alanine aminotransferase (ALT), and **c** alanine phosphatase (ALP). Value represents mean ± SD (n = 7–10), ^*#*^ *p* < 0.05 vs. control group, *p < 0.05 vs. CCl_4_ group

The serum levels of AST, ALT and ALP, the commonly-used biomarkers of liver damage in the clinics [15], were measured by colorimetrical methods. A single dose of CCl_4_ exposure caused severe hepatotoxicity in mice (Fig. 1), the serum levels of AST, ALT and ALP in the CCl_4_ group were dramatically increased by 11.5-fold (46.6 ± 17.8 vs. 610.0 ± 95.6 U/L), 42.2-fold (6.02 ± 3.61 vs. 260.3 ± 60.0 U/L) and 63.1% (101.2 ± 17.3 vs. 165.1 ± 23.8 U/L), respectively, compared with control group. However, these elevations were decreased significantly (p < 0.05) by pre-treatments of PCP at the doses of both 5.15 and 10.3 g/kg BW, and silymarin, a positive control, as well.

### Effects of PCP on CCl4-induced histopathological alteration

**Fig. 2:**
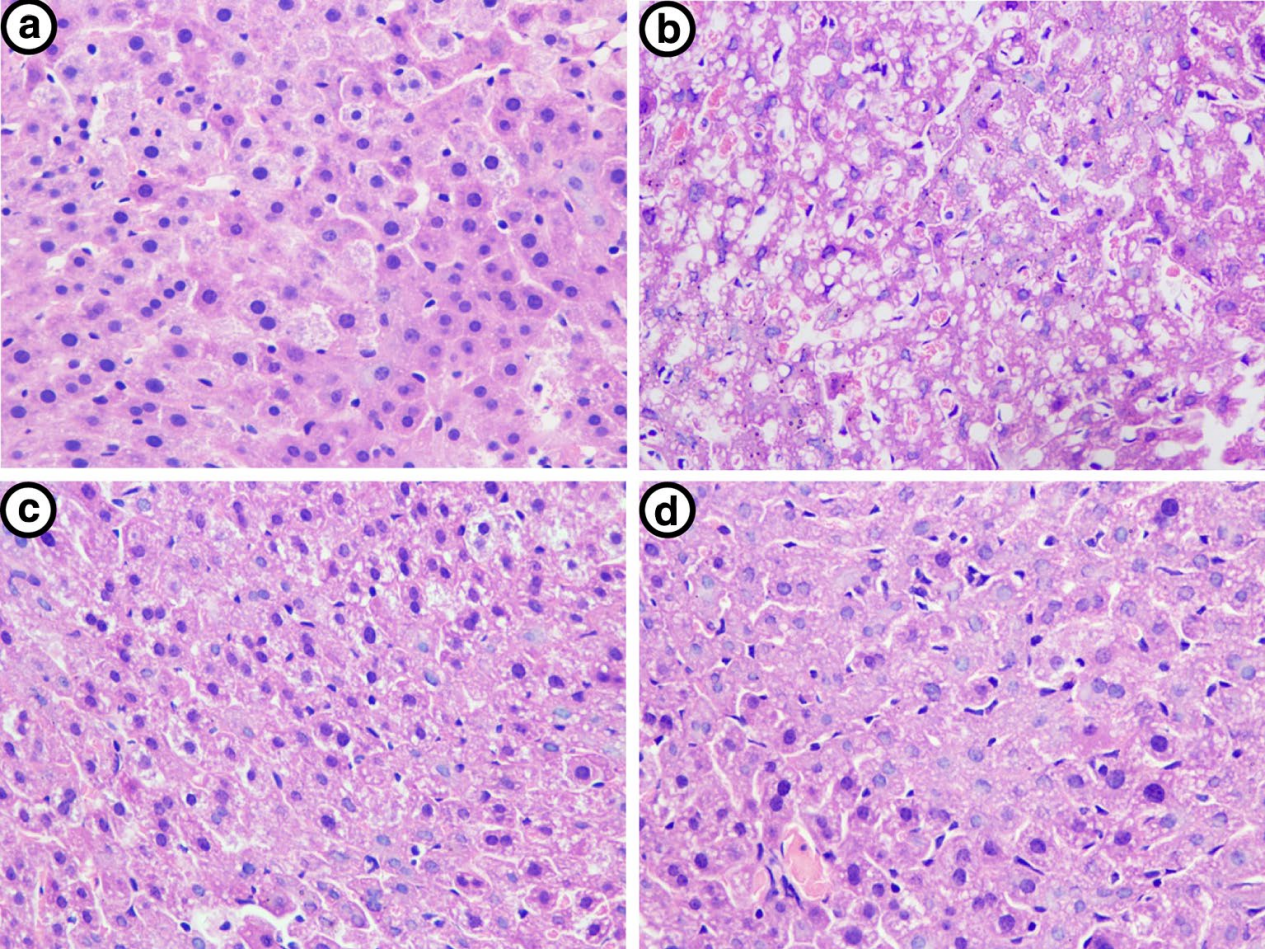
Representative H&E staining of liver tissues. **a** Control, **b** CCl_4_, **c** CCl_4_ + Silymarin (100 mg/kg), and **d** CCl_4_ + PCP (10.3 g/kg BW)

Histological observations were performed to examine the pathological changes in liver. As shown in Fig. 2, the liver tissues from the control group showed normal architectures. CCl_4_ challenge caused marked histopathological changes in the liver, characterized by apparent microvesicular and macrovesicular steatosis, massive inflammatory cells infiltration, and extensive hepatocyte necrosis. These histopathological changes were remarkably ameliorated by the pre-treatments of silymarin and PCP (10.3 g/kg BW), which were consistent with the results of serum parameters.

### Effects of PCP on CCl4-induced hepatocyte apoptosis

**Fig. 3:**
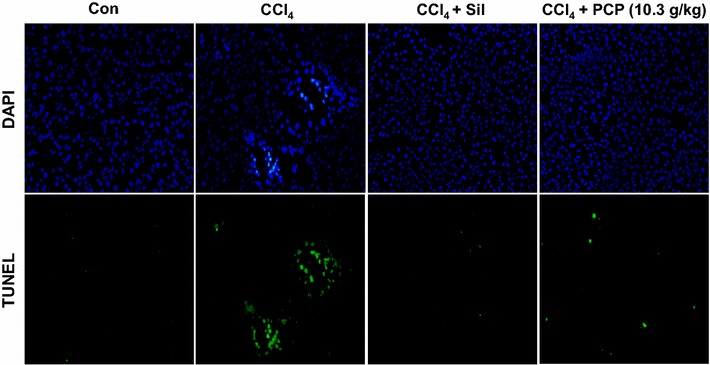
Effects of PCP on CCl_4_-induced hepatocyte apoptosis

As hepatocyte apoptosis also reflects the extent of CCl_4_-induced liver injury [16], a TUNEL assay was performed. As shown in Fig. 3, after 12 h of CCl_4_ challenge, the number of TUNEL-positive cells in liver section was obviously increased over the control group, the apoptotic cells were greatly decreased in the pre-treatment of PCP (10.3 g/kg BW) or silymarin.

### Effects of PCP on CCl4-induced oxidative stress

**Fig. 4:**
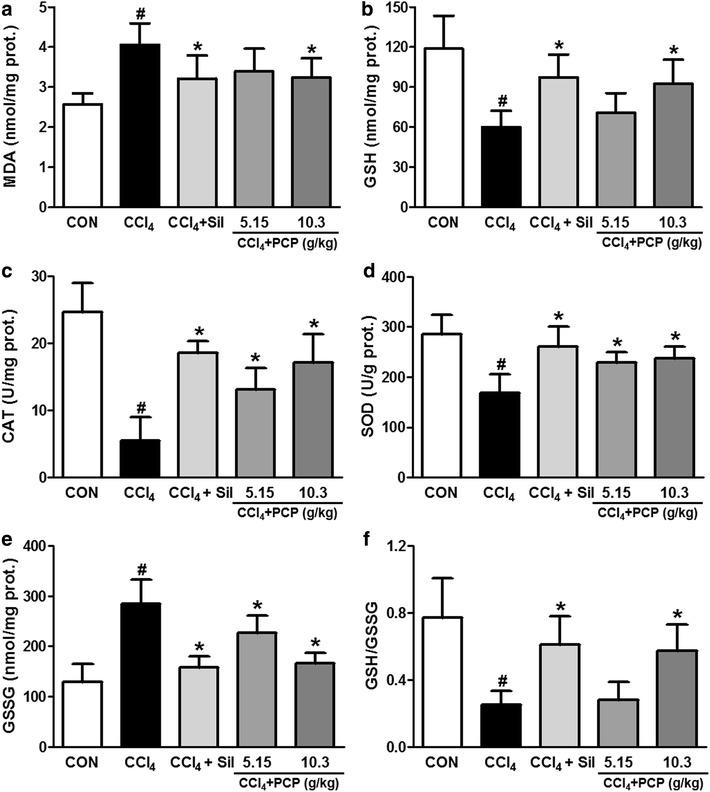
Effects of PCP on CCl_4_-induced oxidative stress in liver. **a** malondialdehyde (MDA); **b** reduced glutathione (GSH); **c** catalase (CAT); **d** superoxide dismutase (SOD); **e** glutathione disulfide (GSSG); **f** the ratio of GSH to GSSG. Value represents mean ± SD (n = 7–10), ^*#*^ *p* < 0.05 vs. control group, *p < 0.05 vs. CCl_4_ group

To assess the protective effects of PCP on hepatic oxidative stress induced by CCl_4_ exposure, the levels of MDA, GSH and GSSG, and the activities of SOD and CAT were examined in liver. As an end-product of lipid peroxidation (LPO), MDA was considered to be a useful marker of oxidative stress [1]. As shown in Fig. 4a, the hepatic MDA level was significantly elevated upon a single dose of CCl_4_ exposure, which was decreased in both silymarin and PCP (10.3 g/kg BW)—treated groups. PCP (5.15 g/kg BW) showed a decreased concentration in hepatic MDA, but with no significant difference. CCl_4_ exposure also depleted endogenous antioxidants, as indicated by the level of GSH, and the activities of SOD and CAT in CCl_4_ group were significantly decreased to 50.5, 22.4 and 58.7%, respectively, compared to control group. These depletions were notably ameliorated by the pretreatment of silymarin and PCP (10.3 g/kg BW) (Fig. 4b–d). Compared to control group, one single dose of CCl_4_ challenge significantly elevated the hepatic GSSG level (129 ± 35 vs. 284 ± 48 n mol/mg protein), leading to the decrease in GSH/GSSG ratio (0.77 ± 0.23 vs. 0.25 ± 0.08) (Fig. 4e, f). These alterations were greatly ameliorated by silymarin (100 mg/kg BW) and PCP (10.3 g/kg BW), and their protective efficacies were comparable.

### Effects of PCP on CYP2E1 expression

**Fig. 5:**
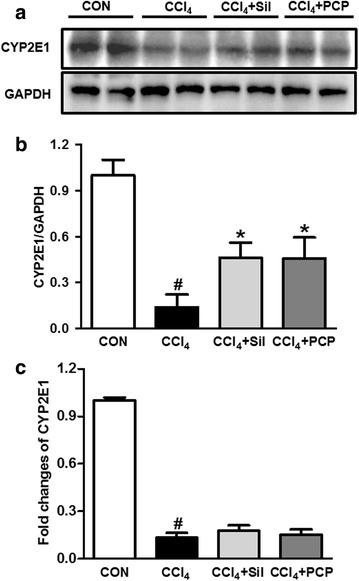
Effects of PCP (10.3 g/kg BW) on CYP2E1 expression. **a** Immunoblot analysis of CYP2E1; **b** its densitometric analysis; **c** qPCR analysis of CYP2E1. Value represents mean ± SD (n = 3–4), ^*#*^ *p* < 0.05 vs. control group, *p < 0.05 vs. CCl_4_ group

As shown in Fig. 5, CCl_4_ challenge dramatically decreased the both mRNA and protein expressions of CYP2E1 in the liver. The pre-treatment of either PCP (10.3 g/kg BW) or silymarin significantly reversed the expression of CYP2E1 in the protein level, but not the transcriptional level.

### Effects of PCP on Nrf2-mediated oxidative stress response pathway

**Fig. 6:**
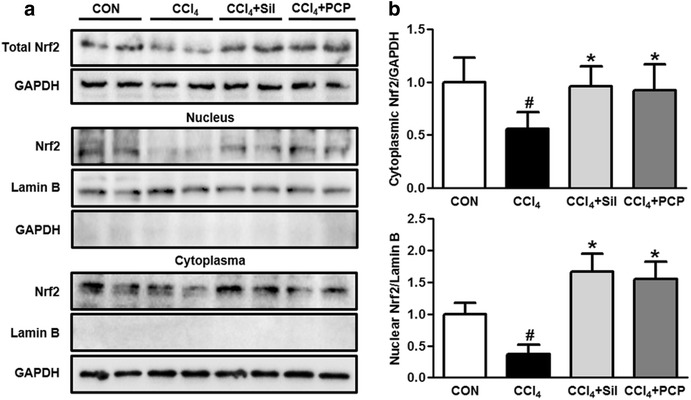
Effects of PCP (10.3 g/kg BW) on protein expression of Nrf2. **a** Immunoblot analysis of Nrf2 in total, nucleus and cytosol; **b** their results of the densitometric analysis. Value represents mean ± SD (n = 3–4), ^*#*^ *p* < 0.05 vs. control group, *p < 0.05 vs. CCl_4_ group

**Fig. 7:**
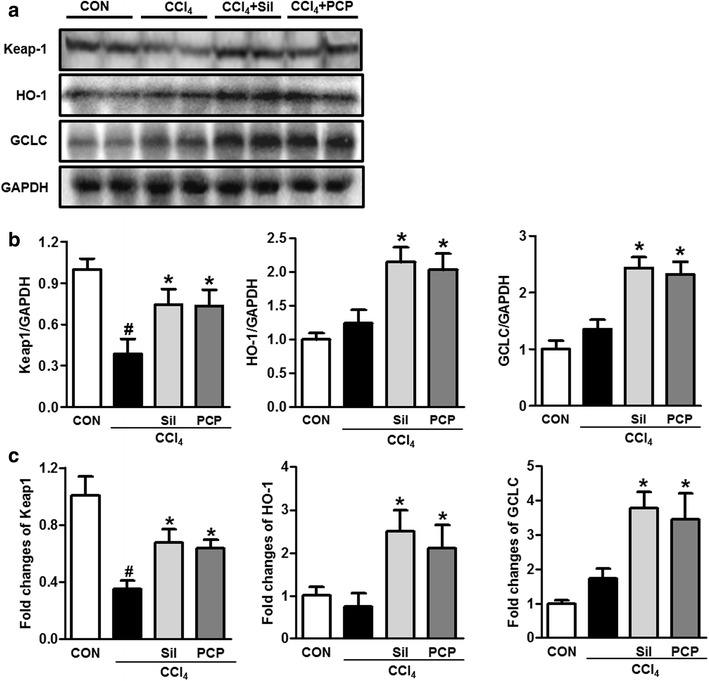
Effects of PCP (10.3 g/kg BW) on Nrf2-mediated oxidative stress response. **a** Immunoblot analysis of Keap1, HO-1 and GCLC; **b** their results of the densitometric analysis; **c** qPCR analysis of Keap1, HO-1 and GCLC. Value represents mean ± SD (n = 3–4), ^*#*^ *p* < 0.05 vs. control group, *p < 0.05 vs. CCl_4_ group

To elucidate the molecular mechanisms underlying the protection of PCP against CCl_4_-induced oxidative stress, the Nrf2 signaling pathway was measured by using immunoblot. Compared to control group, one single dose of CCl_4_ exposure decreased the protein expressions of Nrf2 in total, cytosol and nucleus, the pre-treatment of silymarin and PCP (10.3 g/kg BW) significantly normalized these reductions in Nrf2 expressions induced by CCl_4_ exposure (Fig. 6). Additionally, Keap-1 and the downstream regulated genes of Nrf2 were also examined in the liver by qPCR and immunoblot analysis. As shown in Fig. 7, the pretreatment of silymarin and PCP (10.3 g/kg BW) remarkably increased the expressions of Keap-1, HO-1 and GCLC in terms of both mRNA and protein levels, over the CCl_4_ group. A lower protein expression of Keap-1 in the CCl_4_ group was found compared to the control group. However, no significant difference in either mRNA or protein expression of HO-1 and GCLC was observed between the control and the CCl_4_ groups.

## Discussion

CCl_4_, a well-known hepatotoxicant, has been commonly used in cellular and animal models to evaluate the protective effects of natural products on liver injury [17]. Serum levels of AST and ALT are the preferred indicators for evaluation of liver function, they usually reflect the altered permeability of hepatocellular membrane and the damaged structural integrity of hepatocytes [1]. The elevated ALP level in serum was commonly observed in the patients with extrahepatic, intrahepatic biliary obstruction, and infiltrative liver diseases [18]. In our study, CCl_4_ challenge induced a profound elevation in serum levels of ALT, AST and ALP, indicating the acute hepatotoxicity caused by CCl_4_. However, these increments were effectively alleviated by the pretreatment with PCP and silymarin. The effects of PCP (10.30 g/kg BW) were comparable to silymarin (100 mg/kg BW), a positive control which is a herbal remedy for liver treatment with anti-inflammatory and antioxidant properties [19]. These results demonstrated that PCP effectively protects against acute liver injury induced by a single dose of CCl_4_. This observation was also verified by histopathological examination and TUNEL assay as well.

CCl_4_ is metabolized by CYP2E1 to produce the CCl_4_-derived free radical in the liver [4]. These highly-reactive species can irreversibly oxidize biological macromolecules, such as DNA, proteins and lipids, resulting in lipid peroxidation, oxidative stress, hepatocyte apoptosis, leading ultimately to hepatotoxicity [20, 21]. CCl_4_-induced oxidative stress also depletes the endogenous antioxidants, including the non-enzymatic group, such as GSH, and enzymatic antioxidants, such as SOD and CAT. It was documented that GSH is an important antioxidant in eliminating toxic free radicals and reactive toxic CCl_4_ metabolites [22, 23]. The sulfhydryl residues of GSH molecule is easily oxidized to GSSG, in turn, GSSG can be converted back to GSH by the aid of glutathione reductase (GR). Thus, the redox ratio of GSH/GSSG is often used as a useful indicator of oxidative stress [24]. In this study, CCl_4_ exposure caused severe oxidative stress in the liver where CCl_4_ is primarily metabolized, as evidenced by the increased hepatic levels of MDA and GSSG, the decreased GSH level, GSH/GSSG ratio and antioxidant enzyme activities of CAT and SOD. However, several compounds were identified from PCP, including flavonoids, flavonoid glycosides, polyphenols, steroids [25]. Among these compounds, flavonoids and polyphenols exhibited anti-oxidative effects and other pharmacological bioactivity [26, 27], which may mainly contributed to the hepatoprotection effects of PCP. Our data showed that PCP effectively attenuated CCl_4_-induced oxidative stress by not only reducing hepatic MDA level but also enhancing endogenous non-enzymatic and enzymatic antioxidants.

Hepatic CYP2E1 is mainly responsible for the metabolism of CCl_4_ to produce highly-reactive trichloromethyl free radicals [28]. Thus, CYP2E1 plays a vital role in the regulation of CCl_4_-induced oxidative stress. CYP2E1 deficient mice are resistant to CCl_4_-induced hepatoxicity [29], CYP2E1 inhibitors and the antibodies specific for CYP2E1 reduced liver injury induced by CCl_4_ exposure in rats [30]. While, CCl_4_-induced hepatotoxicity can be enhanced by the pretreatment of alcohol, a CYP2E1 inducer [31]. However, a large body of studies has also indicated that CCl_4_ challenge decreased the expression and activity of CYP2E1 [32–34]. A most plausible explanation is that CCl_4_ might labilize and inactivate CYP2E1, and enhance its degradation, which reveals ongoing oxidative damage [34]. Our data showed that a remarkable decrease in both mRNA and protein expression of CYP2E1 in the liver was observed after CCl_4_ exposure, and the pretreatment of PCP at the doses of either 5.15 or 10.3 g/kg BW significantly elevated the CCl_4_-induced decrease of CYP2E1 expression in the protein level, but not in the transcriptional level, suggesting that PCP efficiently reduces the degradation of CYP2E1 induced by CCl_4_.

To understand how PCP reduces the oxidative stress induced by CCl_4_ exposure, the expressions of Nrf2 and its downstream genes in the liver were measured. Nrf2 plays a critical role in regulating antioxidant defense system in response to oxidative stress [12]. Nrf2-null mice are more susceptible to hepatotoxicity and oxidative stress induced by various chemicals, including CCl_4_, ethanol, acetaminophen, pyrazole and arsenic [35]. In response to stress signals, the redox sensitive protein Keap1 undergoes oxidation, leading to the stabilization of Nrf2 and its nuclear translocation [36]. The activation of Nrf2 by binding to ARE triggers the expression of downstream genes, including HO-1 and GCLC. HO-1 is considered as a strong antioxidant and improves hepatocyte survival. GCLC is a rate-limiting enzyme in GSH biosynthesis in the liver [37]. In the present study, compared with the CCl_4_ group, the hepatic Nrf2 expressions in total, cytosol and nucleus were significantly increased in PCP-treated group. As expected, the PCP-treated group showed higher expressions of Keap-1, HO-1 and GCLC in the liver.

## Conclusion

**Fig. 8:**
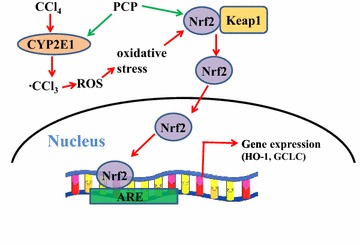
Schematic diagram of the potential mechanisms underlying the protective effects of PCP on the CCl_4_-induced liver injury

Collectively, the pre-treatment of the aqueous extract of PCP (10.3 g/kg BW) can effectively protected against CCl_4_-induced acute liver injury, which was closely similar to efficacy of silymarin (100 mg/kg BW). These hepatoprotective effects might be associated with ameliorating CCl_4_-induced oxidative stress via activation of Nrf2 signaling pathway (Fig. 8).

## Additional file

**Additional file 1.** The Minimum Standards of Reporting Checklist.

## Abbreviations

ALP: alanine phosphatase
ALT: alanine aminotransferase
AST: aspartate aminotransferase
CAT: catalase
CCl_4_: tetrachloride
CFDA: China Food and Drug Administration
CYP2E1: cytochrome P450 2E1
GCL: glutamate cysteine ligase
GR: glutathione reductase
GSH: reduced glutathione
GSH-Px: glutathione peroxidase
GSSG: oxidized glutathione
HO-1: haem oxygenase 1
IVC: individually ventilated cage
Keap1: Kelch-like ECH-associated protein 1
LPO: lipid peroxidation
MDA: malondialdehyde
Nrf2: nuclear factor erythroid 2-related factor 2
PCP: *Penthorum chinense* Pursh
SOD: superoxide dismutase
ROS: reactive oxygen species
